# Dual regulation of the receptor-like kinase *BIR1* involves site-directed transcript cleavage and 5’-leader-mediated translational control

**DOI:** 10.1371/journal.ppat.1014571

**Published:** 2026-09-15

**Authors:** Irene Guzmán-Benito, Carmen Robinson, Livia Donaire, Lourdes Fernández-Calvino, José Manuel Franco-Zorrilla, Ruixia Niu, Guoyong Xu, Catharina Merchante, César Llave

**Affiliations:** 1 Centro de Investigaciones Biológicas Margarita Salas. Consejo Superior de Investigaciones Científicas, Ramiro de Maeztu, Madrid, Spain; 2 Doctorado en Biotecnología y Recursos Genéticos de Plantas y Microorganismos Asociados, Universidad Politécnica de Madrid, Madrid, Spain; 3 Department of Plant Molecular Genetics. Centro Nacional de Biotecnología, Consejo Superior de Investigaciones Científicas, Darwin, Madrid, Spain; 4 State Key Laboratory of Hybrid Rice, Hubei Provincial Research Center for Basic Biological Sciences, Hubei Hongshan Laboratory, RNA Institute, Institute for Advanced Studies (IAS), Wuhan University, Wuhan, Hubei, China; 5 Instituto de Hortofruticultura Subtropical y Mediterránea La Mayora (IHSM-UMA-CSIC), Boulevard Louis Pasteur, Málaga, Spain; China Agricultural University, CHINA

## Abstract

In Arabidopsis, BRASSINOSTEROID INSENSITIVE1-ASSOCIATED RECEPTOR KINASE 1 (BAK1)*-*INTERACTING RECEPTOR-LIKE KINASE 1 (BIR1) is a negative regulator of plant immunity and cell death. *BIR1* was earlier described as a target of epigenetic and post-transcriptional degradation. During virus infections, degradome analysis of *BIR1* transcripts mapped predominant mRNA cleavage sites at the 5’-untranslated leader region (site A) and the protein-coding sequence (sites B and C). Here, we identified another virus-associated cleavage site (D) within the *BIR1* coding region and investigated the contribution of site-directed mRNA cleavage to *BIR1* regulation. Mutations at B, C, and D sites enhanced mRNA stability by impairing transcript cleavage, resulting in increased *BIR1* mRNA and protein accumulation. This regulation is disrupted in RNA silencing mutants, supporting a model of *cis*-directed small interfering RNA (siRNA)-mediated degradation. We next demonstrate that virus infection reduces *BIR1* translation in Arabidopsis. Furthermore, our data reveal a repressive role for the 5’-leader in regulating *BIR1* translation, potentially mediated by upstream open reading frames (uORFs) and a virus-responsive long non-coding RNA (lncRNA) derived from the natural antisense *At4g39838* locus. Together, these findings reveal a multilayered regulatory mechanism that integrates sRNA-mediated cleavage with translational control, with broader implications for the fine-tuning of stress-responsive gene expression during infection.

## Author summary

Plants must tightly control immune responses to ensure effective defense while avoiding immune-associated self-damage. In *Arabidopsis thaliana*, the receptor-like kinase BIR1 functions as a negative regulator of multiple immune resistance pathways. Previous reports have shown that misregulation of *BIR1* leads to cell-death-like phenotypes, underscoring the need for precise control of its expression. However, the mechanisms governing *BIR1* expression have remained largely unexplored. Here, we show that *BIR1* transcripts are subject to post-transcriptional gene silencing and, through mutational analysis, identify the precise cleavage sites involved. In addition, we demonstrate that virus infection compromises *BIR1* translation efficiency and reveal a previously uncharacterized translational control mechanism mediated by the *BIR1* 5’- leader, involving the combined action of multiple upstream open reading frames and a virus-responsive long non-coding RNA derived from the antisense *At4g39838* locus. These findings reveal an unexpected and sophisticated regulatory architecture controlling *BIR1* expression, with potential relevance for the broader *BIR* receptor family and plant immune signaling networks.

## Introduction

Plants have evolved intricate mechanisms to fine-tune the strength and duration of the immune responses, highlighting the importance of immune attenuation to prevent self-damage from prolonged or excessive activation [1,2]. Plant innate immunity comprises pathogen-associated molecular pattern (PAMP)-triggered immunity (PTI) and effector-triggered immunity (ETI) [3]. PTI is mediated by cell surface pattern recognition receptors (PRRs), while ETI involves intracellular nucleotide-binding domain leucine-rich repeat receptors (NLRs) [4]. NLRs are encoded by resistance (*R*) genes and sense pathogen-effector proteins or effector-induced manipulations of host proteins [5,6]. PTI and ETI function synergistically and interdependently in plants and share common downstream responses [7–9]. Besides, ETI often triggers a form of programmed cell death called hypersensitive response (HR) [10].

PTI modulation involves multiple layers, including the formation of PRR-co-receptor complexes, regulation of signal initiation, cytoplasmic signal transduction, and transcriptional control [11–13]. Post-translational mechanisms such as phosphorylation, ubiquitination, and lipid modifications play critical roles in modulating plant immune signaling. Phosphatases like PP2A and PP2C38 attenuate immune responses by regulating the activity of receptor-like kinases (RLKs) BRASSINOSTEROID INSENSITIVE1-ASSOCIATED RECEPTOR KINASE 1 (BAK1) and BOTRYTIS INDUCED KINASE1 (BIK1), respectively [11,14,15]. The ubiquitin-proteasome system controls PRR abundance, as seen in BAK1-mediated activation of E3 ubiquitin ligases (PUB) that target FLAGELLIN-SENSING 2 (FLS2) and BIK1 for degradation [13,16,17]. Additional modifications, including glycosylation and acylation, further influence RLK stability and localization [12]. Finally, MAPK cascades and WRKY transcription factors help balance early PTI signaling through feedback regulation of defense gene expression [11,18].

Similarly, NLR-mediated immune pathways are tightly regulated at multiple levels, including transcriptional, post-transcriptional, translational, and post-translational control. Among these, RNA silencing serves as a key checkpoint modulating both PTI and ETI responses in plants [19,20]. RNA silencing is driven by two major classes of small RNAs (sRNAs): ~ 21-nt microRNAs (miRNAs) and 21–24 nt small interfering RNAs (siRNAs) [21]. Mounting evidence highlights the role of pathogen-responsive miRNAs and siRNAs in plant innate immunity [22–32]. These sRNA networks exert broad post-transcriptional control over NLRs through target RNA cleavage or translational repression, thereby preventing autoimmunity from excessive NLR accumulation [25,33–36]. RNA-directed DNA methylation (RdDM) contributes to immune regulation by silencing transposable elements and nearby defense genes under non-stress conditions [37–39]. Upon pathogen attack, DNA demethylation can lift this repression, activating immune responses and restricting infection [40,41].

The RLK BAK1-INTERACTING RECEPTOR-LIKE KINASE 1 (BIR1) exemplifies another immune regulator whose expression is controlled by both epigenetic and post-transcriptional silencing [42]. *BIR1* is one of the four members of the *BIR* family, of which *BIR1*, *BIR2,* and *BIR3* function to prevent inappropriate activation of BAK1-mediated PRR signaling. Under non-infectious conditions, BIR proteins keep BAK1 inactive through constitutive interaction, but upon pathogen detection, they dissociate, enabling BAK1 to associate with activated PRRs and trigger immune signaling [43–45]. Notably, both *bir1–1* mutants and *BIR1* overexpression lines in Arabidopsis show autoimmune-like phenotypes [42,45], emphasizing the importance of precise *BIR1* regulation. RdDM ensures that *BIR1* expression remains balanced under non-stress conditions [42]. However, upon infection, *BIR1* is transcriptionally induced, likely through a regulatory mechanism involving antagonistic salicylic acid (SA) and jasmonic acid (JA) interactions [46]. Under these conditions, post-transcriptional gene silencing (PTGS) reinforces epigenetic repression to prevent excessive accumulation. This idea is supported by the elevated *BIR1* transcripts in infected RNA silencing mutants and the accumulation of *BIR1*-derived siRNAs specifically in infected plants [42]. Degradome analysis further revealed increased cleavage of *BIR1* in infected tissues, indicating active transcript degradation [42].

In this study, we identified *BIR1* as part of a broad set of plant genes whose mRNAs undergo selective degradation during viral infections but not under control conditions. Mutational analysis of degradome-derived 5’ cleavage sites showed that *BIR1* is post-transcriptionally regulated by RNA silencing-dependent, sequence-specific endonucleolytic cleavage in its coding region. We found that virus-infection interferes with *BIR1* translation, and identified several upstream open reading frames (uORFs) as well as a long non-coding RNA (lncRNA) with complementarity to the TCA-rich region of the *BIR1* 5’-leader, both of which contribute to the regulation of *BIR1* translation. Together, these findings provide new insights into the multilayered mechanism governing *BIR1* expression.

## Results

### *BIR1* transcripts undergo endonucleolytic cleavage in virus-infected plants

**Fig 1:**
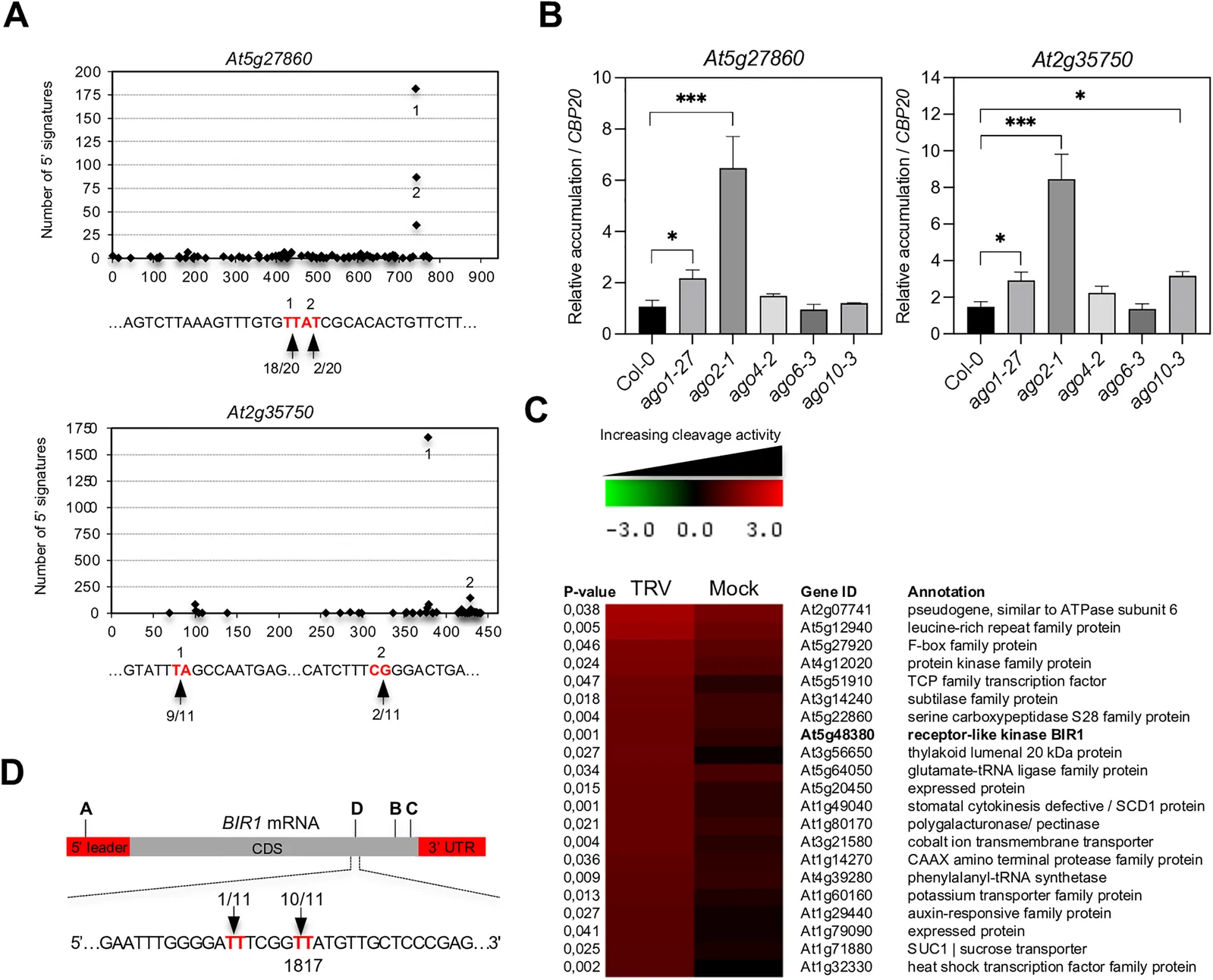
Genome-wide identification of degradation RNA targets using a Parallel-analysis of cDNA Ends (PARE)-based strategy. **(A)** Target plots derived from degradome sequencing showing 5’ cleavage signatures along the *At2g35750* and *At5g27860* mRNAs. Prominent peaks indicate preferential cleavage sites. Cleavage positions validated by 5’-RACE are shown below the plots and labeled with the corresponding numbers; clone counts indicate relative cleavage frequency. **(B)** RT-qPCR analysis of *At2g35750* and *At5g27860* transcript levels in wild-type plants (Col-0), slicer-competent ARGONAUTE (AGO) mutants (*ago1-27* and *ago2-1*), RNA-Directed DNA Methylation (RdDM)-associated, non-slicing AGO mutants (*ago4-2*, *ago6-3*) and the miRNA-related *ago10-3* mutant. Transcript levels were normalized to the *CBP20* internal control. Data represent mean ± SD (n = 3) and were analyzed by one-way ANOVA followed by Tukey’s multiple-comparison test. **(C)** Heatmap showing Arabidopsis genes with the highest levels of differential RNA degradation between mock- and tobacco rattle virus (TRV)-infected plants, identified by PARE analysis followed by Affymetrix ATH1 microarray profiling. The color scale represents log2(TRV/Mock) ratios; corresponding p-values (F-test, df = 1, 4) are indicated. *BIR1* (*At5g48380*) is highlighted in bold. **(D)** *BIR1* transcript cleavage sites identified by 5’-RACE in TRV-infected plants. Clone counts indicate cleavage frequency. Site D marks the most frequently detected cleavage position; additional cleavage sites (A-C) are also shown [42]. No 5’-RACE products were detected in mock-inoculated controls.

In a previous study, degradome profiling of uncapped and cleaved transcripts identified specific cleavage hotspots within *BIR1* transcripts from tobacco rattle virus (TRV)-infected Arabidopsis plants, indicating that *BIR1* undergoes endonucleolytic processing during infections [42]. To explore this further, we employed a genome-wide strategy to identify candidate RNA silencing targets in mock-inoculated and TRV-infected Arabidopsis plants. This approach uses parallel analysis of RNA ends (PARE) to generate libraries containing 3’ cleavage mRNA fragments, followed by Affymetrix ATH1 microarray hybridization of enriched cDNAs [47]. Among the transcripts producing significant amounts of cleaved fragments relative to the hybridization controls, two genes (*At2g35750* and *At5g27860*) showing the highest fold enrichment under both conditions were selected for further validation as RNA silencing targets (S1 Table). RNA-seq-based degradome profiling, together with gene-specific 5’-RACE, confirmed specific and recurrent cleavage at defined sites in both transcripts (Fig 1A). In each case, the 5’-RACE products precisely matched the cleavage positions identified by the degradome data. Moreover, relative to wild-type plants (WT, Col-0), transcript levels of both genes were significantly increased in Arabidopsis mutants defective in slicer-competent AGO proteins (*ago1–27* and *ago2–1*), but not in mutants affecting RdDM-associated, non-slicing AGOs (*ago4–2*, *ago6–3*) (Fig 1B) [48]. Accumulation of *At2g35750*, but not *At5g27860*, transcripts increased in the *ago10–3* mutant allele. This suggests that *At2g35750* regulation also involves AGO10, which functions as a miRNA decoy in Arabidopsis [49]. These results confirm that this approach reliably identifies *bona fide* targets of post-transcriptional RNA silencing. We then identified 47 and 98 genes with significantly enriched 3’ RNA cleavage products in mock-inoculated and TRV-infected plants, respectively (S1 Table). As expected, we found that *BIR1* transcripts also accumulated significantly more 3’ degradation products in TRV-infected plants (*p* < 0.05) than in mock-treated controls, confirming that *BIR1* transcripts were selectively cleaved during viral infection (Fig 1C, S1 Table). Previously, degradome analysis mapped cleavage sites within the *BIR1* transcript to nucleotide positions 156, 2,219, and 2,247, designated as sites A, B, and C, respectively [42]. Using gene-specific 5’-RACE, we identified an additional virus infection-associated cleavage site at position 1,817 (designated D; Fig 1D), distinct from those found by degradome sequencing [42]. This finding further supports the selective degradation of *BIR1* transcripts in response to TRV infection. None of the experimentally identified cleavage sites within the *BIR1*-coding region matched annotated miRNAs in the miRBase database.

### Mutations within the predicted cleavage sites enhance *BIR1* expression

**Fig 2:**
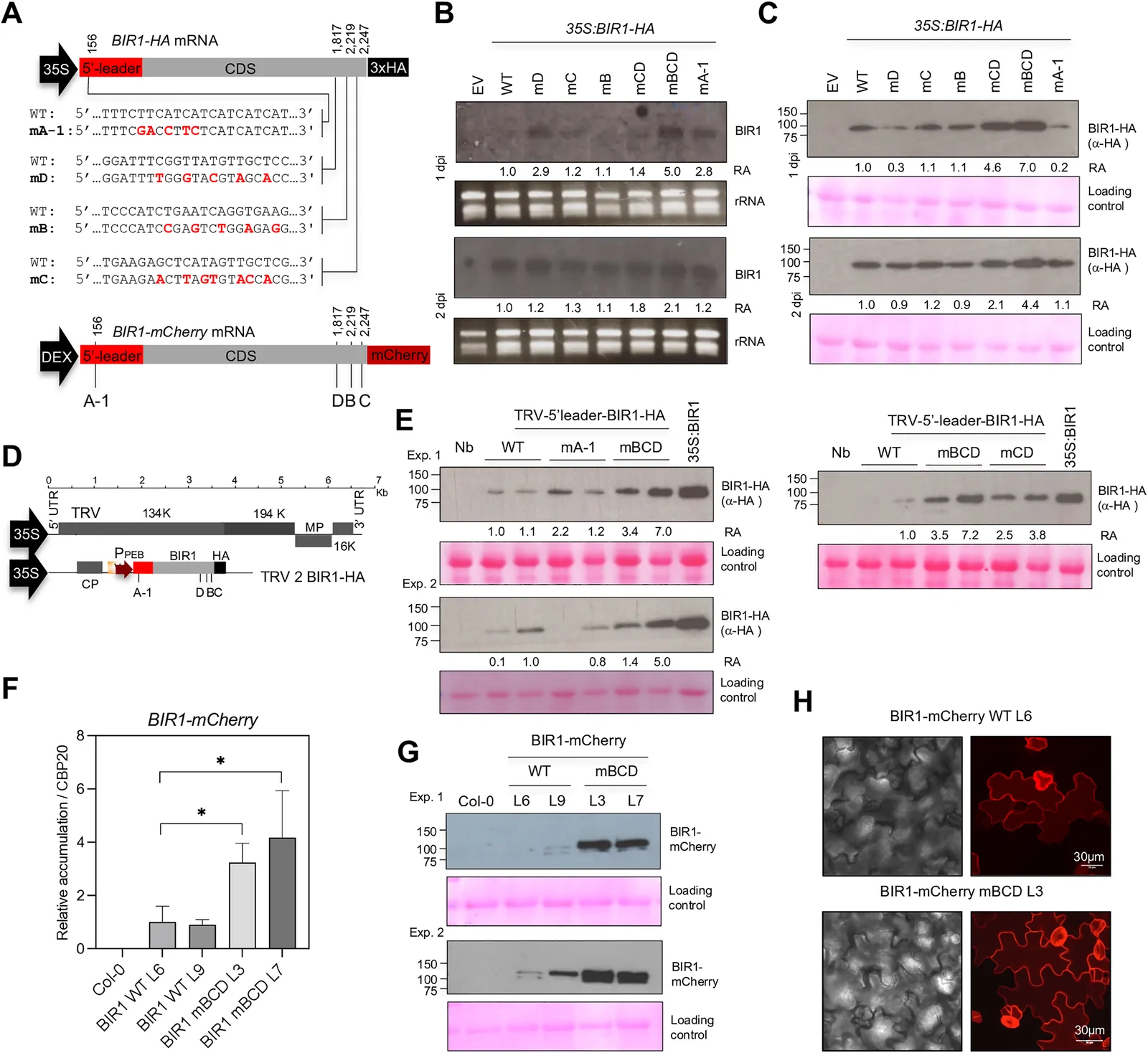
Effect of mutations at B, C and D cleavage sites within the protein-coding region on *BIR1* expression. **(A)** Top, schematic of 35S-driven constructs expressing HA-tagged BIR1 variants. Mutations at cleavage sites A-D (nucleotide positions indicated) mutations are shown in red; sites B-D were altered by synonymous substitutions. Bottom, schematic of DEX-inducible constructs expressing mCherry-tagged wild-type (WT) or mutant variants of *BIR1*. **(B)** RNA blot of WT and mutant *BIR1* (mA-1, mB, mC, mD, mCD, mBCD) transcripts from the 35S-driven constructs shown in panel A (top), sampled at 1- and 2-days post-infiltration (dpi). Blots were hybridized with *BIR1* cDNA probes and normalized to ethidium bromide staining. Empty vector (EV) is the negative control. **(C)** Western blot of HA-tagged WT and mutated BIR1 proteins, collected at 1 and 2 dpi. **(D)** Schematic of tobacco rattle virus (TRV)-based constructs expressing HA-tagged BIR1 variants from the pea early browning virus (PEBV) replicase promoter in pTRV2. Transcription of pTRV1 and pTRV2 is 35S-driven. Cleavage sites A1-D are marked. **(E)** Western blots of HA-tagged WT and mutated BIR1 (left: mA-1 and mBCD; right: mBCD and mCD) from TRV-infected *Nicotiana benthamiana* leaves, sampled at 5 days post-inoculation (dpi). A 35S-driven BIR1-HA construct served as control. In left, Results from two independent experiments (Exp. 1 and 2) are shown. **(F)** RT-qPCR of *BIR1-mCherry* transcript levels in DEX-treated (16 h, 30 µM) Arabidopsis lines expressing WT (L6, L9) or mutated mBCD (L3, L7) *BIR1-mCherry*. Transcript levels were normalized to the *CBP20* internal control. Data represent mean ± SD (n = 3) and were analyzed by one-way ANOVA followed by Tukey’s multiple-comparison test. *p < 0.05. **(G)** Western blot of mCherry-tagged BIR1 proteins in DEX-treated (4 days) transgenic lines expressing WT (L6, L9) and mutated mBCD (L3, L7) *BIR1-mCherry*. Non-transgenic Col-0 served as a negative control. Results from two independent experiments (Exp. 1, Exp. 2) are shown. **(H)** Confocal images of mCherry fluorescence in DEX-treated (4 days) transgenic lines expressing WT (L6) and mBCD (L3) BIR1-mCherry. Scale bar = 30 µm. Imaging used a Leica TCS SP5 confocal microscope (561 nm excitation). All *BIR1* constructs shown comprise the 5’ untranslated leader region followed by the *BIR1* coding sequence (CDS). Ponceau staining shows protein loading. Protein standards (KDa) are indicated. Relative accumulation (RA) shown with WT set to 1.0. Experiments were independently repeated with consistent results.

To determine the effects of single cleavage sites for *BIR1* mRNA regulation, we generated a panel of *BIR1* constructs carrying silent mutations at each of the B, C, or D positions within the *BIR1* open-reading frame (Fig 2A, top). Both the WT and mutated constructs included the 5’-leader and a C-terminal triple human influenza hemagglutinin (3xHA) tag, and were transiently expressed in *N. benthamiana* under the 35S promoter. *BIR1* mRNA and protein levels were assessed at 1- and 2-days post-infiltration (dpi) by Northern and Western blot, respectively. RNA blots revealed that individual mutations (mB, mC, or mD) exerted only modest and variable effects on *BIR1* transcript accumulation across independent experiments, whereas the combined mutation of all three *BIR1* cleavage sites (mBCD) consistently increased transcript abundance relative to WT (Fig 2B). Accordingly, BIR1 proteins translated from cleavage-resistant mCD or mBCD transcripts were also more abundant than those from WT transcripts or transcripts with single mutations (Fig 2C).

To rule out agroinfiltration-related artifacts affecting *BIR1* expression, we further evaluated the impact of mBCD mutations using a viral-expression system. HA-tagged *BIR1* WT, mCD and mBCD constructs, including their native 5’-leader, were cloned into the pTRV2 vector and co-delivered with pTRV1 into *N. benthamiana* via Agrobacterium infiltration (Fig 2D). *BIR1* expression was then analyzed in upper, non-infiltrated, systemically-infected leaves. As observed previously in the agroinfiltration assay, Western blot analysis showed higher levels of BIR1-HA protein derived from mCD or mBCD transcripts compared to WT in these leaves (Fig 2E). TRV genomic RNA levels were comparable across WT, mCD, mBCD, and green fluorescence protein (GFP) controls in systemic leaves, indicating that the differences in *BIR1* expression were not due to variations in virus accumulation (S1A Fig).

We further generated mCherry-tagged versions of the *BIR1* WT and the triple mutant (mBCD), including the 5’ untranslated leader region, that were conditionally expressed in Arabidopsis using a transgenic dexamethasone (DEX)-inducible system (Fig 2A, bottom) [42]. Samples from two independent lines were collected after treatment with 30 µM DEX. RT-qPCR showed higher accumulation of mutated transcripts (BIR1 mBCD L3 and L7) compared to WT (BIR1 WT L6 and L9), indicating that these mutations confer cleavage resistance and thereby increase mRNA stability (Fig 2F and S1B Fig). Consistently, BIR1 protein translated from mBCD transcripts in DEX-inducible L3 and L7 lines was more abundant than protein from WT transcripts in L6 and L9 transgenic lines (Fig 2G). These findings also confirmed that the observed effect was not tag-dependent. Both BIR1-mCherry WT and mBCD proteins localized to the plasma membrane, ruling out altered subcellular localization as a cause of increased mBCD accumulation (Fig 2H). Collectively, our results from multiple expression systems and plant species establish that sequence alterations at cleavage sites within the *BIR1* coding region consistently enhance *BIR1* expression.

**Fig 3:**
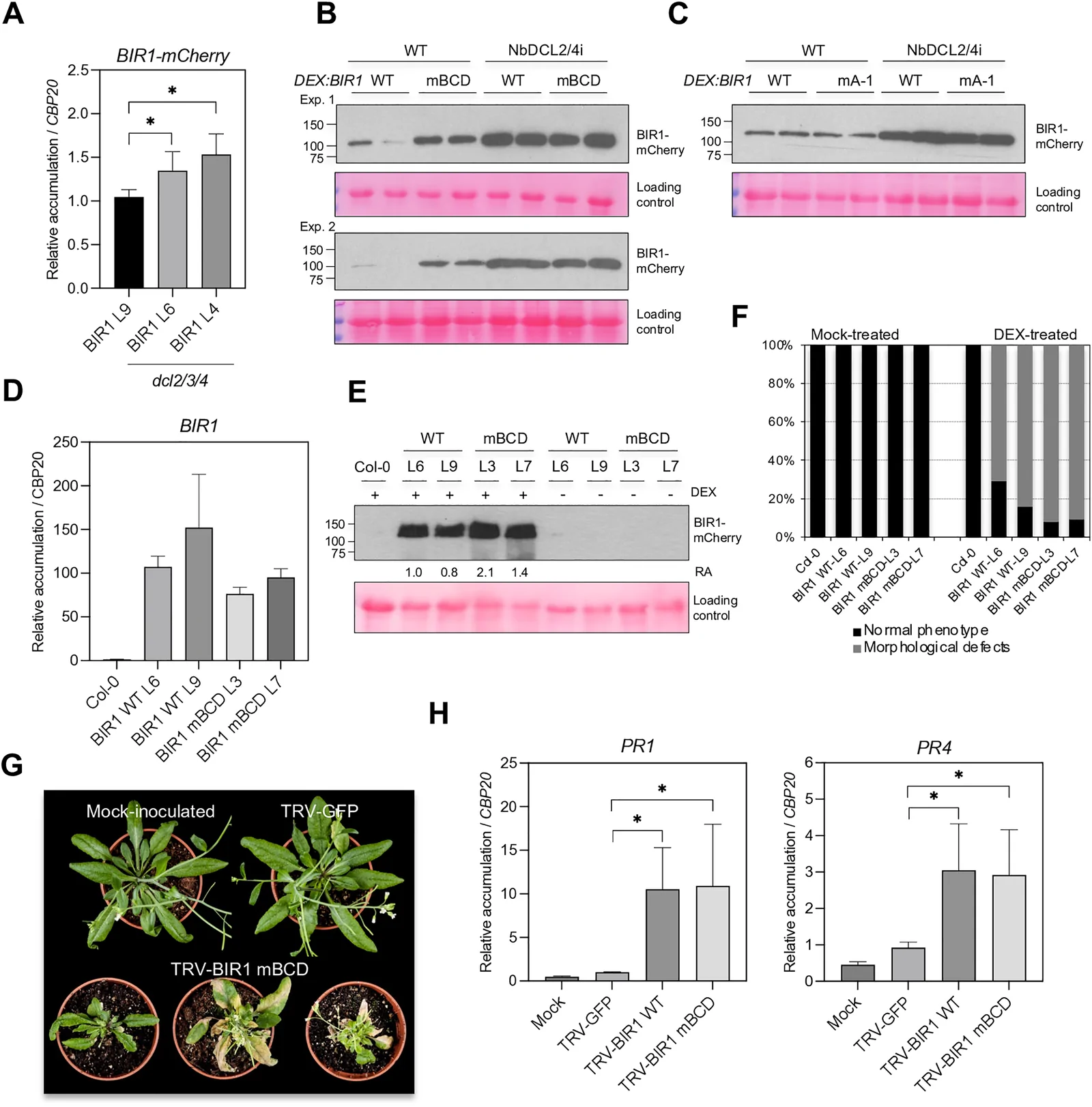
*BIR1* overexpression interferes with site-directed transcript regulation. **(A)** RT-qPCR of *BIR1-mCherry* transcript levels in dexamethasone (DEX)-inducible transgenic plants of Col-0 (L9) and *dcl2 dcl3 dcl4* (*dcl234*; L4, L6) genotypes expressing wild-type (WT) *BIR1-mCherry*, 24 h after DEX treatment (30 µM) (n = 5). **(B)** Western blot of mCherry-tagged proteins WT and mBCD BIR1 in *Nicotiana benthamiana* WT and NbDCL2/4i leaves, 2-day post-infiltration (dpi) after DEX induction. Results from two independent experiments (Exp. 1, Exp. 2) are shown. **(C)** Western blot of mCherry-tagged WT and mA-1 BIR1 in *N. benthamiana* WT and NbDCL2/4i, as in **(B)**. Two samples per construct/genotype are shown. **(D)** RT-qPCR analysis of *BIR1-mCherry* transcripts in Arabidopsis lines expressing WT (L6, L9) or mutant (mBCD; L3, L7) *BIR1-mCherry*. Plants were treated with DEX (+) or H2O (-) for 10 days (n = 5). **(E)** Western blot of mCherry-tagged WT and mBCD BIR1 proteins in the same lines as in (C) after 10 days of DEX or H2O treatment. Non-transgenic Col-0 served as a negative control. Relative accumulation (RA) shown with WT (L6) set to 1.0. **(F)** Percentage of plants with normal or altered morphology in WT (L6, L9) and mBCD (L3, L7) Arabidopsis lines after DEX treatment. Non-transgenic Col-0 and H2O-treated plants were used as controls. **(G)** Morphology of soil-grown plants mock-inoculated, infected with tobacco rattle virus (TRV)-green fluorescence protein (GFP), or TRV expressing BIR1 mBCD. Images taken at 14 dpi. **(H)** RT-qPCR analysis of *PATHOGENESIS-RELATED 1* (*PR1*), and *PR4* transcripts in plants from **(G)** (n = 5). Mock-inoculated, and TRV-GFP-infected plants served as controls. Transcript levels were normalized to the *CBP20* internal control. Data represent mean ± SD and were analyzed by one-way ANOVA followed by Tukey’s multiple-comparison test. *p < 0.05. Immunoblots used anti-mCherry antibodies; Ponceau staining shows loading controls. *BIR1* constructs shown comprise the 5’ untranslated leader region followed by the *BIR1* coding sequence (CDS). All experiments were repeated with consistent results.

The accumulation of *BIR1*-derived siRNAs and the detection of discrete cleavage sites, particularly during viral infection, strongly suggests that *BIR1* expression is regulated by post-transcriptional gene silencing [42]. To determine whether cleavage depends on RNA silencing, we generated Arabidopsis lines expressing *BIR1-mCherry* under a DEX-inducible promoter in the *dcl2 dcl3 dcl4* (herein *dcl234*) mutant background defective in sRNA biosynthesis and silencing amplification. RT-qPCR analysis showed higher levels of transgenic *BIR1-mCherry* transcripts in two independent *dcl234* lines (L4 and L6) compared with the BIR1 L9 control (Fig 3A), consistent with the increased accumulation of endogenous *BIR1* transcript observed previously in RNA silencing mutants [42]. Likewise, transient expression assays in *N. benthamiana* revealed higher accumulation of the BIR1-mCherry mBCD protein in WT plants, whereas this difference was abolished in NbDCL2/4i plants, in which sRNA-mediated cleavage is impaired (Fig 3B). In contrast, mCherry-tagged *BIR1* constructs carrying the mA-1 mutation in the 5’ leader accumulated to comparable protein levels in both genetic backgrounds **(Fig 3C)**, indicating that regulation of site A differs from that of sites B, C, and D. This finding further supports the idea that RNA silencing-associated cleavage at sites B, C, and D regulate *BIR1* expression.

### *BIR1* overexpression compromises its post-transcriptional regulation

We further explored whether site-directed transcript cleavage alone was sufficient to contain the expression of *BIR1* in the absence of transcriptional regulation. To test the hypothesis, we used the DEX-inducible system to overexpress the mBCD mutated version of *BIR1-mCherry* in independent transgenic lines (BIR1 mBCD L3 and L7). Overexpression of *BIR1* induces cell death phenotypes and triggers transcriptional responses that greatly overlap with those of canonical PTI and ETI [50]. RT-qPCR showed that daily DEX applications for 10 days resulted in elevated transcript levels of both WT and mBCD compared to mock-treated controls (Fig 3D). Western blot analysis revealed slightly higher levels of BIR1 protein derived from mBCD transcripts compared with the WT at this time point (Fig 3E). Overexpression of *BIR1*-mBCD led to developmental and morphological defects characteristic of *BIR1* overexpression, appearing between 10 and 12 days after induction, similar to what was observed with the WT version [42]. Interestingly, a slightly higher proportion of BIR1-mBCD transgenic plants exhibited these morphological defects following DEX treatments, suggesting that increased protein levels resulting from the loss of site-directed cleavage in the mBCD mutant may enhance the manifestation of cell death-associated morphologies (Fig 3F). Cell death and induction of the defense marker genes *PATHOGENESIS-RELATED 1* (*PR1)* and *PR4*, reported upon *BIR1* WT overexpression [42], were also observed when mBCD *BIR1* was expressed via the TRV vector in Arabidopsis (Fig 3G, H) [42]. These findings indicate that high-level transcriptional activation of *BIR1* overrides the effects of site-directed transcript regulation. Thus, while sequence-specific cleavage normally contributes to constraining *BIR1* expression under physiological conditions, its regulatory impact appears to be attenuated when *BIR1* is strongly overexpressed, possibly due to saturation or reduced efficiency of the RNA silencing machinery.

### *BIR1* translation is influenced by regulatory elements within its 5’-leader

**Fig 4:**
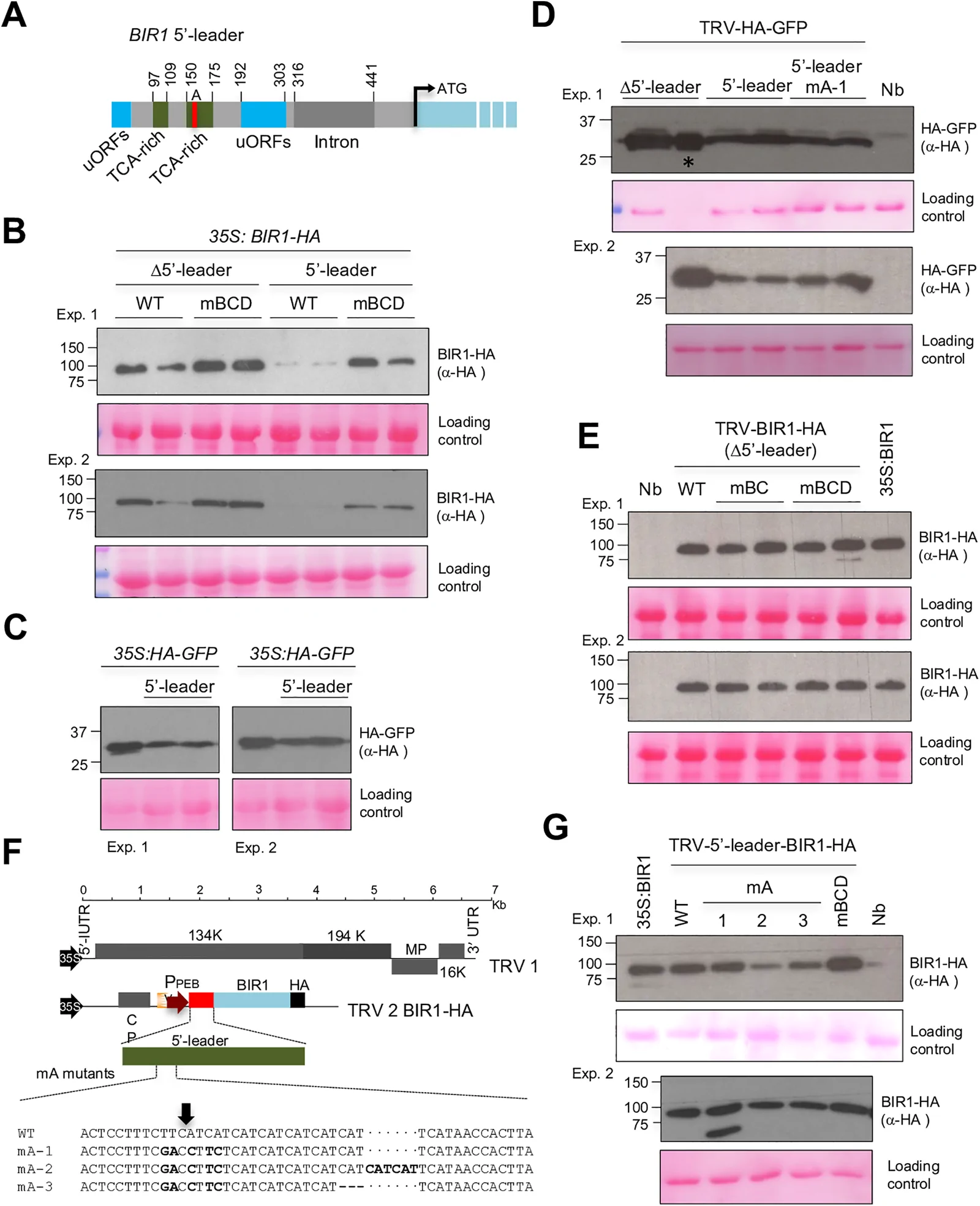
Effects of the 5’ leader region on *BIR1* translation efficiency. **(A)** Schematic of *cis*-regulatory elements within the 5’-leader of *BIR1*. The location and coordinates of upstream open reading frames (uORF), TCA-rich repeats and intron are shown. The canonical ATG start codon is shown. Cleavage site A is indicated in red. **(B)** Western blot comparing HA-tagged BIR1 expression with or without the 5’-leader, using 35S-driven WT and mBCD BIR1 constructs in the transient assay at 2-day post-infiltration (dpi). **(C)** Western blot comparing HA-tagged GFP expression with or without the 5’-leader using 35S-drive constructs in the transient assay at 2 dpi. **(D**) Western blot comparing HA-tagged GFP expression with or without the 5’-leader (WT and mutated mA-1) via TRV in *Nicotiana benthamiana*, sampled at 5 days post-inoculation (dpi). Results from two independent experiments (Exp. 1 and 2) are shown. The asterisk (*) indicates a 4x diluted sample. **(E)** Western blot of HA-tagged WT and mutated BIR1 (mBC, mBCD) lacking the 5’-leader expressed from tobacco rattle virus (TRV) constructs, sampled at 5 dpi. Results from two independent experiments (Exp. 1 and 2) are shown. **(F)** Schematic of tobacco rattle virus (TRV)-based constructs expressing HA-tagged wild-type (WT) or mutated *BIR1* (mA variants). Constructs include the *BIR1* 5’-leader and coding sequence (CDS), driven by the pea early browning virus (PEBV) replicase promoter in pTRV2. Transcription of pTRV1 and pTRV2 is 35S-driven. Cleavage sites A-D are marked; synonymous mutations are shown in Fig 2A. The major A site at position 156 (identified by degradome sequencing) is marked with a thick arrow. **(G)** Western blot of HA-tagged WT and mA mutant BIR1 proteins (mA-1, mA-2, mA-3) from TRV-infected *N. benthamiana* leaves (constructs from **A)**, sampled at 5 dpi. A 35S-driven BIR1-HA construct served as control. Results from two independent experiments (Exp. 1 and 2) are shown. All immunoblots used anti-HA antibodies; Ponceau staining shows protein loading. Protein standards (KDa) are indicated. Experiments were independently repeated with consistent results.

The 5’-leader of plant mRNAs is instrumental in controlling mRNA stability, translation efficiency, and responsiveness to developmental or environmental cues [51,52]. The *BIR1* 5’-leader is a highly complex regulatory region that contains TCA-rich domains, putative sRNA-binding sites, and multiple upstream open reading frames (uORFs), all of which have the potential to influence translational regulation **(Fig 4A)**. Thus, we examined the role of the 5’-leader in *BIR1* translation by expressing several 35S-driven *BIR1*-coding constructs, with or without the 5’-leader sequence, using agroinfiltration in *N. benthamiana*. Constructs containing the 5’-leader produced lower levels of BIR1 protein compared to those without it, suggesting a repressive effect (Fig 4B). To test this further, we designed chimeric constructs expressing HA-tagged enhanced GFP (eGFP) from transcripts with or without the *BIR1* 5’ leader. In both 35S-driven expression and TRV-mediated delivery in *N. benthamiana*, transcripts with the 5’-leader led to reduced eGFP protein accumulation (Fig 4C, D). These results suggest that the 5’-leader region contains regulatory elements that negatively modulate translation. Noteworthy, when *BIR1* constructs lacking the 5’-leader were expressed via agroinfiltration (Fig 4B) or delivered via TRV (Fig 4E) in *N. benthamiana*, differences in WT and mBCD protein accumulation were attenuated, suggesting that site-specific cleavage, and the resulting differences in expression, are also influenced by elements located within the 5’-leader. We therefore examined the role of regulatory elements within the 5´-leader of *BIR1* in modulating its expression.

### Mutations at or near cleavage site A have no effect on *BIR1* expression

Cleavage site A was consistently identified as a major degradation hotspot in degradome libraries from Arabidopsis leaves and inflorescences [42]. This site, along with nearby secondary cleavage sites, maps within the TCA-repeat-rich region of the *BIR1* 5’-leader sequence [42]. We then explored the role of the cleavage site A and additional downstream sequences in regulating *BIR1* expression. HA-tagged *BIR1* WT and mA-1 mutant were transiently expressed in *N. benthamiana* under the 35S promoter (Fig 2A). Northern and Western blot analyses showed that the mA-1 mutation had little to no effect on *BIR1* mRNA and protein levels (Fig 2B, C), consistent with TRV-mediated expression of HA-tagged *BIR1* mA-1 (Fig 2E) and with the transient expression of a mCherry-tagged *BIR1* mA-1 mutant under a DEX-inducible promoter (Fig 3C). Likewise, mutating cleavage site A (mA-1) within the *BIR1* 5’-leader had no detectable effect on its ability to repress translation of the eGFP reporter (Fig 4D). We next generated two additional *BIR1* mutants, an insertion mutant (mA-2) and a deletion mutant (mA-3), targeting sequences downstream of site A, expressed using TRV as a viral vector (Fig 4F). Western blot analysis of systemically infected leaves revealed similar HA-tagged BIR1 protein levels in WT and mA-1, with modest reductions in mA-2 and mA-3. As expected, the mBCD control showed higher BIR1 accumulation than WT or any mA variant (Fig 4G). These findings suggest that mutations at or near site A have limited effect on *BIR1* expression *in vivo*. Comparable TRV levels across samples confirm that differences in *BIR1* expression were not due to infection variability (S1A Fig).

### The *BIR1* leader contains multiple translated uORFs

**Fig 5:**
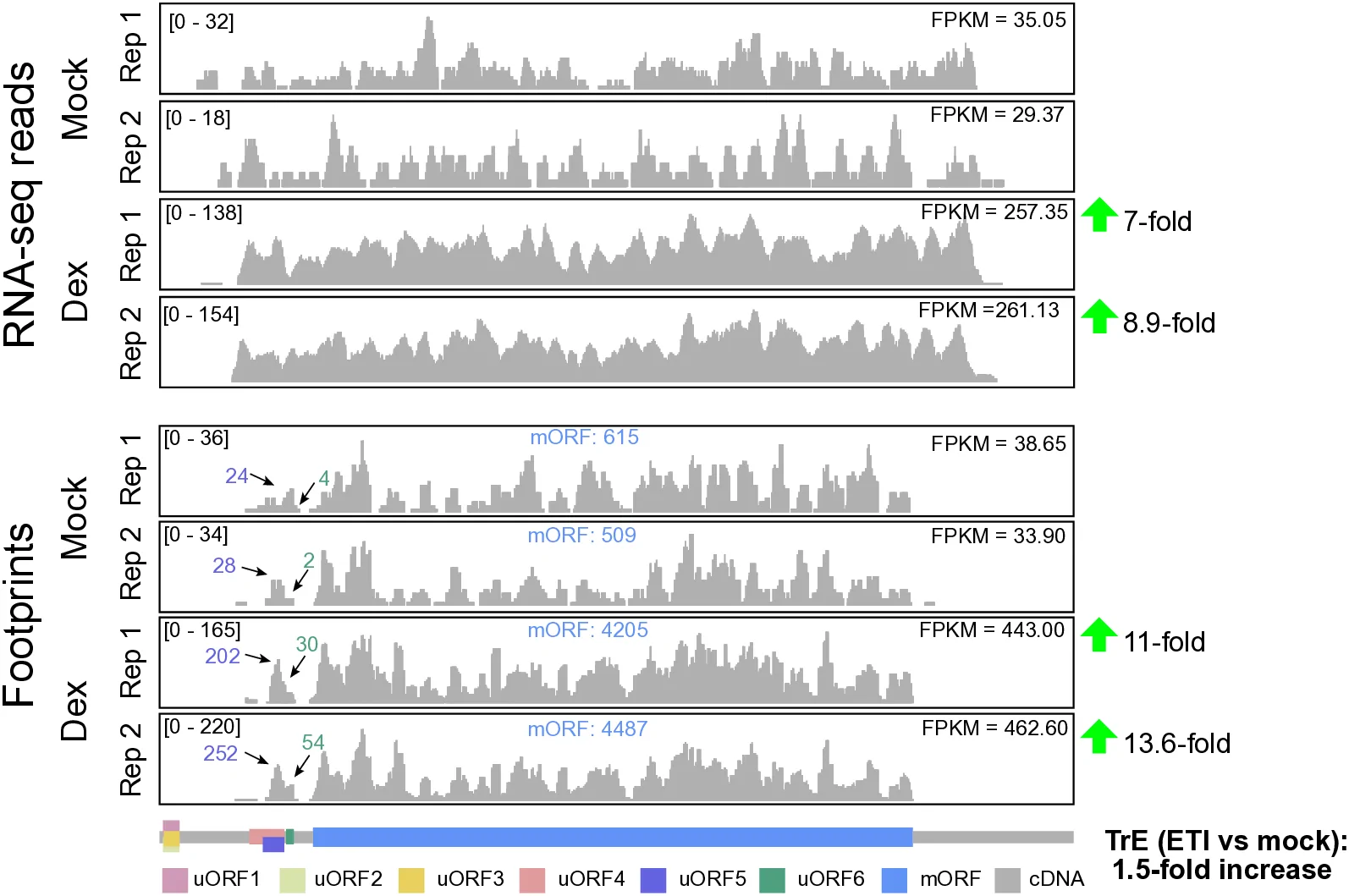
Translational control of *BIR1* expression involved multiple upstream open reading frames. Normalized distributions of RNA-seq and Ribo-seq reads along the *BIR1* transcript in Col-0 plants expressing a dexamethasone (Dex)-inducible AvrRpt2 effector from *Pseudomonas syringae* under mock and ETI-induced conditions. Transcript expression levels are given as Fragments Per Kilobase of transcript per Million mapped reads (FPKM). The grey bar represents the *BIR1* cDNA, with color-coded boxes indicating the six annotated upstream open reading frames (uORFs) and the main open reading frame (mORF). Numbers above the read profiles indicate read counts for uORF5, uORF6, and the mORF. Numbers following the green arrows denote fold changes in transcript and ribosome footprint abundance upon DEX treatment. The fold change in translational efficiency (TrE), representing the ratio of Ribo-seq to RNA-seq signals, is shown in bold. Data from [57].

uORFs, which are small ORFs located within the 5´-leader sequences upstream of the main ORF (mORF), can act as molecular switches, activating or mostly repressing mORF translation in response to developmental and environmental cues [53,54]. According to the TAIR11 transcript annotation and uORFlight database [55], *BIR1* harbors six uORFs in its 5’-leader (Fig 5). In TAIR11, the transcription start site (TSS) is defined using the longest annotated 5′-end [56]. However, inspection of RNA-seq read coverage revealed that transcripts initiating from this longest 5′-end are rare. Consequently, the first three annotated uORFs are present only in low-abundance alternative TSS isoforms, whereas the majority of *BIR1* transcripts lack these three uORFs (Fig 5).

Therefore, our subsequent analyses focused on the three uORFs that are consistently present in the predominant transcript isoforms (uORFs 4, 5, and 6). To investigate whether these uORFs are translated and contribute to the regulation of *BIR1* expression, we analyzed publicly available Ribo-seq data from Arabidopsis Col-0 plants [57]. Normalized Ribo-seq and RNA-seq reads mapped along the *BIR1* transcript revealed ribosome footprints at all three uORFs, indicating that these *BIR1* uORFs are recognized by translating ribosomes (Fig 5). Noteworthy, uORFs translation also occurs under ETI-induced conditions, as observed in Arabidopsis plants expressing a DEX-inducible version of AvrRpt2, an effector protein secreted by pathogenic *Pseudomonas syringae* (Fig 5). Consistent with previous reports [42], ETI induction resulted in a marked increase in both *BIR1* mRNA abundance (~8-fold) and translation (~12-fold), meaning that ETI enhanced the translation efficiency (TrE) of *BIR1* by approximately 1.5-fold (Fig 5). To assess whether this increase in translation could be attributed to altered translation dynamics of the uORFs, we compared the ratios of total Ribo-seq footprint reads in the mORF versus the uORFs, as well as between uORF5 and uORF6, under both mock and DEX-induced conditions. These analyses revealed no significant differences that could account for the observed increase in mORF TrE (Fig 5). However, given that these uORFs, particularly uORFs 5 and 6, are recognized by ribosomes and translated, they represent strong candidates for regulating *BIR1* translation.

### A long non-coding RNA (lncRNA) modulates *BIR1* translation

**Fig 6:**
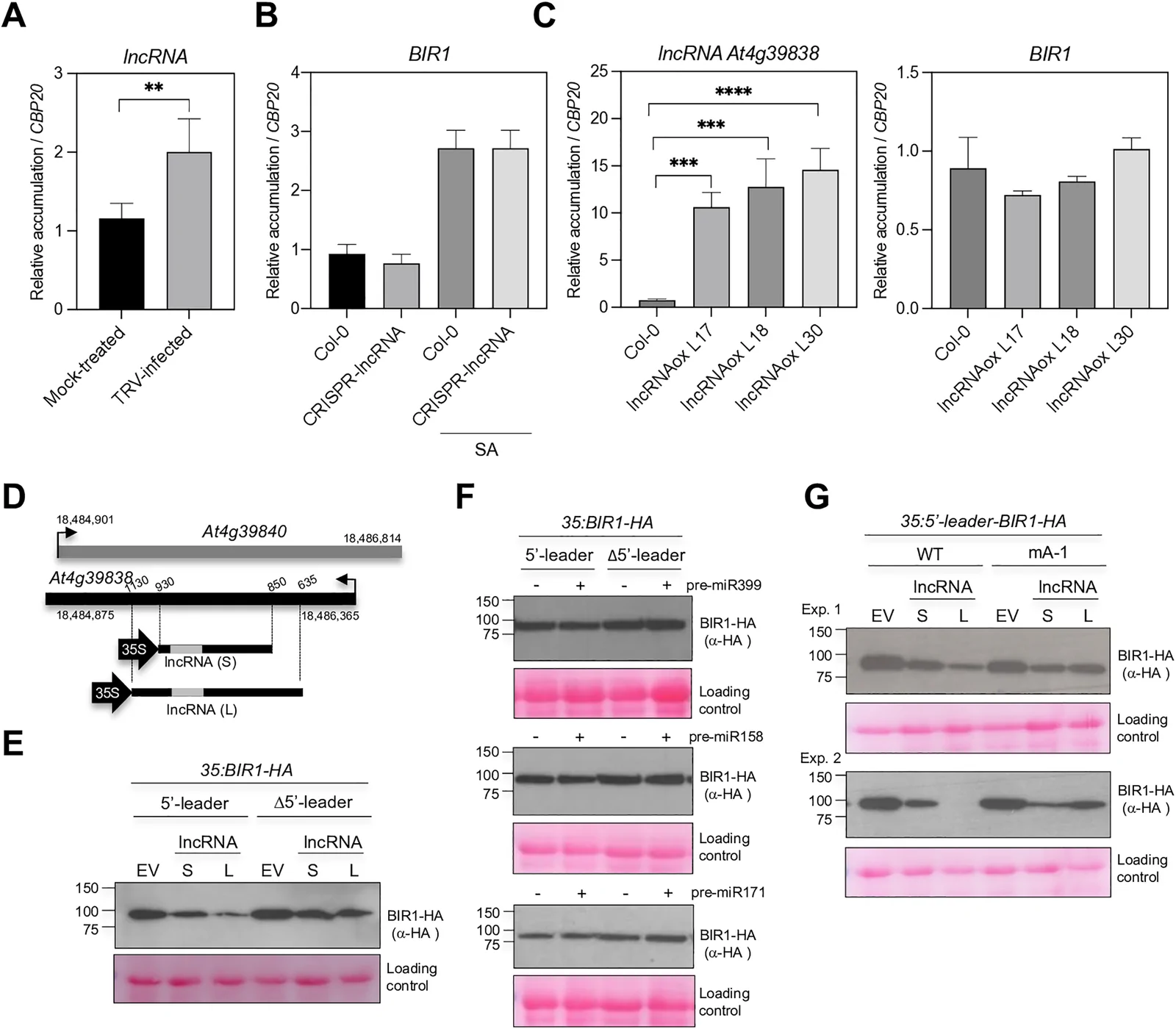
Effects of the *At4g39838*-derived lncRNA on *BIR1* transcript and protein accumulation. **(A)** RT-qPCR analysis of long non-coding (lncRNA) transcript levels in tobacco rattle virus (TRV)-infected Arabidopsis relative to mock-treated plants at 5 days post-inoculation. **(B)** RT-qPCR analysis of *BIR1* transcript levels in the CRISPR-Cas9-*At4g39838* knockout line. Levels were measured in mock-treated and salicylic acid (SA)-treated plants. **(C)** RT-qPCR of lncRNA (left) and *BIR1* (right) transcript levels in transgenic Arabidopsis lines (L17, L18, L30) overexpressing the *At4g39838* locus under the 35S promoter in Arabidopsis. Transcript levels were normalized to the *CBP20* internal control. Data represent mean ± SD (n = 4-6) and were analyzed by one-way ANOVA followed by Tukey’s multiple-comparison test. ****p < 0.0001, ***p < 0.001, **p < 0.01. **(D)** Diagram of the overlapping natural antisense pair *At4g39838* and *At4g39840*, with transcription start sites indicated by arrows and genome coordinates shown. 35S-driven constructs express long (L) and short (S) sequence versions of the *At4g39838*-derived lncRNA. The predicted sequence interacting with the TCA-rich region of the *BIR1* 5’-leader is shown in grey. **(E)** Western blot of HA-tagged BIR1 in leaves co-infiltrated with lncRNA (S or L) and 35S-driven WT BIR1 constructs, with or without the 5’-leader. **(F)** Western blot of HA-tagged BIR1 in leaves co-infiltrated with 35S-driven lncRNAs harboring Arabidopsis miR399, miR158, or miR171 precursors, and WT *BIR1* constructs, with or without the 5’-leader. Controls (-) were infiltrated with empty vector (EV). **(G)** Western blot of HA-tagged BIR1 in leaves co-infiltrated with lncRNA (S or L) and WT or mutated (mA-1) *BIR1* constructs containing the 5’-leader. EV-infiltrated leaves served as controls. Samples were collected at 2-day post-infiltration (dpi). Immunoblots were probed with anti-HA antibody; Ponceau staining shows loading. Protein standards (KDa) are indicated. Experiments were independently repeated with consistent results.

LncRNAs can regulate translation through base pairing with the 5’ untranslated region (5’ UTR) of target genes [58,59]. We therefore searched the Arabidopsis genome for non-protein-coding loci with sequence complementarity to the *BIR1* 5’-leader and identified the natural antisense transcript *At4g39838*, which overlaps the stress-responsive gene *At4g39840*. Notably, *At4g39838* shares partial complementarity with the TCA-rich region of the *BIR1* 5’-leader. We hypothesized that *At4g39838* functions as a regulatory lncRNA, with the TCA repeats serving as its functional binding site. Notably, *At4g39838*-derived lncRNA transcripts accumulated significantly in TRV-infected Arabidopsis plants relative to mock-treated controls, suggesting a potential role in *BIR1* regulation during infection (Fig 6A). To investigate its effect on *BIR1* transcript accumulation, we first genotyped two Arabidopsis T-DNA insertion lines (SALK_093172C and SALK_097507C) targeting the overlapping *At4g39838*/*At4g39840* region. Both insertions were located upstream of the predicted lncRNA-binding sequence (S**2A Fig**). RT-PCR analysis showed only a slight reduction in *At4g39838* transcript levels in homozygous mutants relative to WT plants, suggesting that regulatory activity was not abolished in these lines (S2B Fig). We next generated a homozygous CRISPR–Cas9 Arabidopsis line carrying a 698-bp deletion in the *At4g39838* locus that disrupted the predicted *BIR1* binding site within the lncRNA. RT-qPCR analysis revealed comparable *BIR1* transcript levels in WT and knockout plants under both basal conditions and after salicylic acid (SA) treatment (Fig 6B). Likewise, constitutive transgenic overexpression of a 35S-driven *At4g39838* construct in Arabidopsis did not alter *BIR1* transcript accumulation (Fig 6C). Collectively, our data indicated that *At4g39838* does not affect *BIR1* transcript stability or abundance.

To determine whether *At4g39838* regulates *BIR1* at the translational level, we generated two 35S-driven constructs containing the predicted lncRNA sequence complementary to the *BIR1* 5’-leader, either alone (S, short) or together with its flanking sequences (L, long). These constructs were co-expressed with a 35S-driven HA-tagged *BIR1* containing the 5’-leader by agroinfiltration in *N. benthamiana* (Fig 6D). Strikingly, both *At4g39838*-derived lncRNA transcripts caused a strong decrease in BIR1 protein accumulation compared to the empty vector (Fig 6E). As expected, this effect was greatly reduced when a *BIR1* construct lacking the 5’-leader was used, indicating that this region was required for *At4g39838*-mediated repression of *BIR1* expression (Fig 6E). Moreover, co-expression of unrelated lncRNA harboring miRNA precursors (miR399, miR158, miR171) had no effect on BIR1 protein levels, regardless of the presence of the 5’-leader, confirming that the *At4g39838*-derived lncRNA operates specifically on regulatory elements within the 5’-leader of *BIR1* transcripts (Fig 6F). Repression also occurred when the lncRNA was co-expressed with a *BIR1* mA-1 construct, indicating that site A mutations in our study were not sufficient to prevent lncRNA-mediated regulation (Fig 6G). Together, these results demonstrate that the *At4g39838*-derived lncRNA exerts a sequence-specific, 5’-leader-dependent regulatory effect on *BIR1* translation, and identify the TCA-rich region as a potential regulatory element within the 5’-leader of *BIR1*.

### Identification of potential site A-interacting sRNAs in Arabidopsis

Comparison of *BIR1* degradome signatures with miRBase identified the annotated miRNA ath-miR5658 as a near-perfect match to cleavage site A (S2C Fig). ath-miR5658 also exhibits partial complementarity to downstream sequences in the *BIR1* 5’leader, where redundant cleavage events were previously detected by PARE and 5’-RACE (S2C Fig) [42]. Interestingly, genomic analysis revealed that this putative miRNA maps to the *At4g39838*-derived lncRNA locus, in a region predicted to fold into a characteristic stem-loop (hairpin) precursor structure (S2D Fig). ath-miR5658 expression was reported to be reduced in *dcl1* mutant alleles and detected in multiple Arabidopsis tissues by RT-qPCR [60]. However, in our study, we were unable to detect either the mature or passenger strands by sRNA blotting, stem-loop RT-qPCR, or sRNA library sequencing in WT plants or silencing mutants. Moreover, overexpression of the predicted precursor in *N. benthamiana* failed to yield detectable sRNA signals (S2E Fig), or canonical miRNA-associated effects. Although the basis for the discrepancy with the previous report by [60] remains unclear, it may arise from differences in genetic background (accession-specific variations), detection sensitivity, or stress conditions that promote overlapping siRNAs production over canonical miRNA biogenesis, as previously shown [61,62]. Virus infection increased both sense and antisense virus-associated siRNAs (vasiRNAs) at this locus, suggesting that ath-miR5658-like sequences may originate from both overlapping *At4g39838/At4g39840* dsRNA intermediates and ath-miR5658-containing hairpins, rather than from a single biogenesis trigger (S3A Fig). As shown above, neither depletion nor overexpression of the *At4g39838* locus in Arabidopsis affected *BIR1* transcript levels*,* suggesting a minimal, if any, contribution of the ath-miR5658 to transcript cleavage under our experimental conditions (Fig 6B, C). We next cross-referenced degradome sequencing data with theoretically predicted Arabidopsis sRNA datasets, identifying a candidate with near-perfect complementarity to site A and adjacent positions within the TCA-rich region (S2F Fig). Similar to ath-miR5658, cleavage at site A mapped precisely to the tenth nucleotide of this sRNA, consistent with canonical slicing. This sRNA matches with five Arabidopsis genes (S2G Fig), including *BASIC REGION/LEUCINE ZIPPER MOTIF 60* (*bZIP60; At1g42990)*, which produced abundant vasiRNAs in virus-infected but not mock-treated plants (S3B Fig). The lack of stem-loop potential at this locus supports its classification as a vasiRNA, however, there is currently no experimental evidence supporting the existence or functionality of this sRNA.

### Virus infection reduces *BIR1* translational efficiency in Arabidopsis

**Fig 7:**
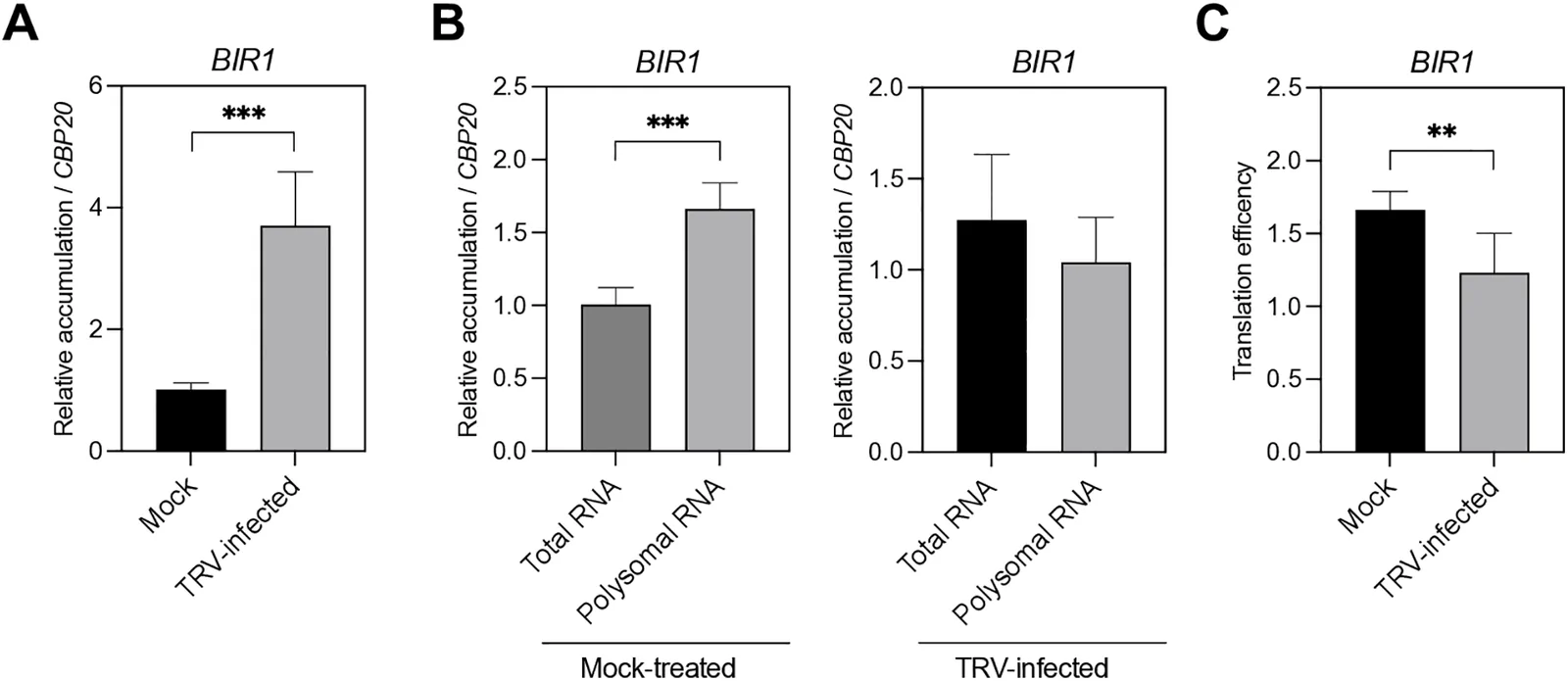
Tobacco rattle virus (TRV) infection increases endogenous *BIR1* transcript accumulation while reducing translational efficiency. **(A)** RT-qPCR analysis of *BIR1* in mock-treated and TRV-infected plants at 5 days post-inoculation (dpi). **(B)** RT-qPCR analysis of *BIR1* in total and polysomal RNA fractions from mock-treated (left) and TRV-infected (right) plants. **(C)** Translational efficiency of *BIR1*, calculated as the ratio of transcript abundance in polysomal RNA relative to total RNA. Transcript levels were normalized to the *CBP20* internal control. Data represent mean ± SEM (mock, *n* = 5; TRV-infected, *n* = 7) and were analyzed using unpaired t-test with Welch’s correction. ***p < 0.001, **p < 0.01.

Given that lncRNAs can regulate translation, that the *At4g39838*-derived lncRNA is induced during viral infection, and that its co-expression reduces *BIR1* expression, we investigated whether *BIR1* is translationally regulated during TRV infection. Total and polysomal RNA fractions were isolated from mock-treated and TRV-infected Arabidopsis plants to compare *BIR1* transcript abundance. As expected, total *BIR1* mRNA accumulated approximately fourfold following TRV infection (Fig 7A). Under mock conditions, *BIR1* transcripts were significantly enriched in the polysomal fraction, indicating preferential association with actively translating ribosomes (Fig 7B). In contrast, this enrichment was lost upon TRV infection. We found that despite the marked increase in total *BIR1* transcript levels, polysome-associated *BIR1* mRNA did not increase proportionally. Consequently, the translational efficiency of *BIR1*, estimated as the ratio of polysomal to total mRNA abundance, was significantly reduced in infected plants compared with mock-treated controls (Fig 7C). These results indicate that TRV infection uncouples *BIR1* transcript accumulation from translation, supporting a role for translational regulation in controlling *BIR1* expression during viral infection.

## Discussion

RNA silencing plays a key role in regulating endogenous genes under various stress conditions. For instance, during viral infection, vasiRNAs derived from endogenous genes accumulate and likely guide the silencing of their gene of origin [63,64]. Here, we identified novel candidate RNA silencing targets in Arabidopsis by detecting increased accumulation of cleaved 3’ mRNA products in virus-infected plants compared to mock controls. Among them, the receptor-like kinase *BIR1* emerges as a virus-responsive gene undergoing selective degradation. Previous work and our current results indicate that post-transcriptional silencing contributes to *BIR1* regulation during viral infection [42]. Notably, activation of *BIR1* silencing in response to TRV does not correlate with increased expression of canonical RNA silencing genes (S2 Table) [65]. Using 5’-RACE, we identified a novel cleavage site (D) in *BIR1* transcripts that complements two previously described degradome-supported sites (B and C) [42]. Together, these sites define preferential cleavage hotspots within the *BIR1* coding sequence. Simultaneous mutation of sites B, C, and D increased *BIR1* mRNA and protein accumulation in both Arabidopsis and *N. benthamiana* across three independent expression systems carrying either HA or mCherry C-terminal tags, indicating that these sites contribute to transcript degradation. In contrast, single-site mutations generally produce weaker effects, suggesting that all three sites contribute collectively to *BIR1* degradation. The accumulation of *BIR1*-derived vasiRNAs during infection, together with the increased abundance of *BIR1* transcripts in DCL-defective plants, supports a model in which cleavage at sites B, C, and D is mediated by *cis*-acting siRNAs [42]. However, we were unable to identify the specific siRNA(s) responsible for cleavage through degradome-supported target pairing, likely due to insufficient sequencing depth in our sRNA libraries. The B, C and D mutations were designed to generate cleavage-resistant *BIR1* transcripts, although we cannot exclude the possibility that they also alter local RNA structure and thereby enhance transcript stability independently of cleavage. However, this explanation appears unlikely, as the mutations consistently produced similar effects on *BIR1* accumulation in constructs carrying different C-terminal tags, whose transcripts are expected to differ substantially in both local and global transcript folding. Together, these observations support a model in which the mutations reduce the accessibility of cleavage sites to AGO/vasiRNA-mediated slicing.

Interestingly, post-transcriptional cleavage fails to contain *BIR1* expression when the transcriptional regulation is lost, and the gene is highly transcribed over time, as in our DEX-inducible system. This suggests that cleavage supports transcriptional regulation functioning as a fine-tuning mechanism to maintain *BIR1* transcript levels within a functional threshold. Notably, cleavage sites B and C are located 40 and 13 nucleotides upstream of the stop codon, respectively, a region often associated with ribosome pausing and co-translational decay [66,67]. The 28-nt spacing between B and C matches the footprint of a stalled ribosome [68], raising the possibility that ribosome pausing near the stop codon may contribute to the observed cleavage patterns. However, this model does not fully explain why synonymous mutations at these sites increase *BIR1* transcript and protein levels, even when using HA- or mCherry-tagged versions. Alternative explanations, such as sequence-specific cleavage by other endonucleases in infected tissue, cannot be ruled out.

We found that cleavage at site A, together with redundant secondary cleavages in the *BIR1* 5’ leader, aligns with two Arabidopsis sRNAs. However, neither candidate sRNA was recovered in our sRNA libraries, and degradome-supported alignments alone are insufficient to demonstrate functionality. ath-miR5658 was previously reported to target the promoter region of *At3g25290,* thereby enhancing transcript accumulation in Arabidopsis [60]*.* In or study, however, ath-miR5658 does not appear to contribute significantly to *BIR1* transcript regulation. Likewise, the predicted *bZIP60*-derived vasiRNA remains supported only by *in silico* analyses, with no experimental evidence for its biogenesis to date. Site A represents a strong degradome signal under normal conditions, suggesting biological relevance. Unexpectedly, however, mutations disrupting site A had no detectable effects on *BIR1* expression. This observation may reflect functional redundancy among cleavage sites across the TCA-enriched region or indicate that regulation occurs only under specific physiological conditions in Arabidopsis, not reproduced in our heterologous systems. Alternatively, cleavage at site A may not itself constitute a regulatory event, but instead arise as a downstream consequence of impaired *BIR1* translation. Further studies will be required to distinguish between these possibilities and determine whether cleavage at site A is mediated by biologically active sRNAs or by sRNA-independent mechanisms.

A key finding of our study is that the translational efficiency of *BIR1* is dynamically modulated during immune responses. In ETI-induced plants, ribosome footprinting indicates a modest increase in *BIR1* translational efficiency, whereas polysome pelleting of TRV-infected plants revealed a significant reduction. Although these estimates were obtained using different experimental approaches and metrics and therefore cannot be quantitatively compared, the reduction in translational efficiency observed during TRV infection is not apparent in the AvrRpt2 dataset. This suggests that translational repression of *BIR1* may require infection-associated signals beyond ETI activation. We also cannot exclude the possibility that other pathogens, including bacteria acting independently of ETI, similarly modulate *BIR1* translation. Collectively, these findings identify translational control as an additional regulatory layer contributing to the context-dependent regulation of *BIR1* during immune responses. In this context, we found that the *BIR1* 5’ leader acts as a translational repressive element, likely involving both sequence and structural features [69,70]. We identified six type 1 uORFs within the 5’-leader, three of which (uORFs 4–6) are actively translated. uORFs are well-stablished *cis*-regulatory elements that can suppress downstream mORF translation through mechanisms such as ribosome stalling, leaky scanning, or inefficient reinitiation [71–73]. In plants, uORF-mediated translational control is widespread among immune- and stress-responsive genes and can be regulated during pathogen infection and stress [54]. Here, analysis of Ribo-seq data from transgenic Arabidopsis revealed active translation of the *BIR1* uORFs under both mock and ETI conditions [57], supporting a potential regulatory role for these elements. One possibility is that ribosomes initiating at upstream uORFs subsequently reinitiate at the *BIR1* mORF, leading to coordinated increases in ribosome occupancy on both regions following ETI activation. Alternatively, given their efficient translation, the uORFs may exert regulatory functions through their encoded peptides, which could act *in trans* to modulate *BIR1* translation or signaling pathways [74]. Further mutational analyses targeting individual uORFs will be required to unequivocally establish their precise contribution to *BIR1* translational regulation, particularly during viral infections.

lncRNAs are known to regulate translation by interacting with RNA elements within the 5’ UTR [69]. We identified a virus-inducible lncRNA derived from the *At4g39838* locus that represses *BIR1* translation without affecting transcript accumulation. This finding suggests that the lncRNA contributes, at least in part, to the reduced translation efficiency of *BIR1* during viral infection. Given that *BIR1* acts as a negative regulator of immunity, induction of the lncRNA during viral infection could transiently repress *BIR1* translation while allowing transcripts to accumulate. These stored mRNAs could then be rapidly recruited to ribosomes following pathogen clearance, enabling swift restoration of BIR1 protein levels to dampen immune signaling and re-establish cellular homeostasis. The effect of this lncRNA depends on the *BIR1* 5’ leader region and appears to involve sequence complementarity, as other lncRNAs carrying miRNA precursors have no effect on *BIR1* expression, supporting a specific RNA-RNA interaction. Although interference with other regulatory motifs remains possible, we proposed that the TCA-rich region within the *BIR1* 5’ leader serves as a complementary binding site for the lncRNA. The molecular basis of this repression, however, remains unresolved, as direct sequence-specific base pairing between defined regions of the *At4g39838*-derived lncRNA and the *BIR1* 5’-leader has not yet been demonstrated. Elucidating the mechanism of this interaction, as well as its contribution to the translational regulations observed during viral infections, represents an important direction for future research.

**Fig 8:**
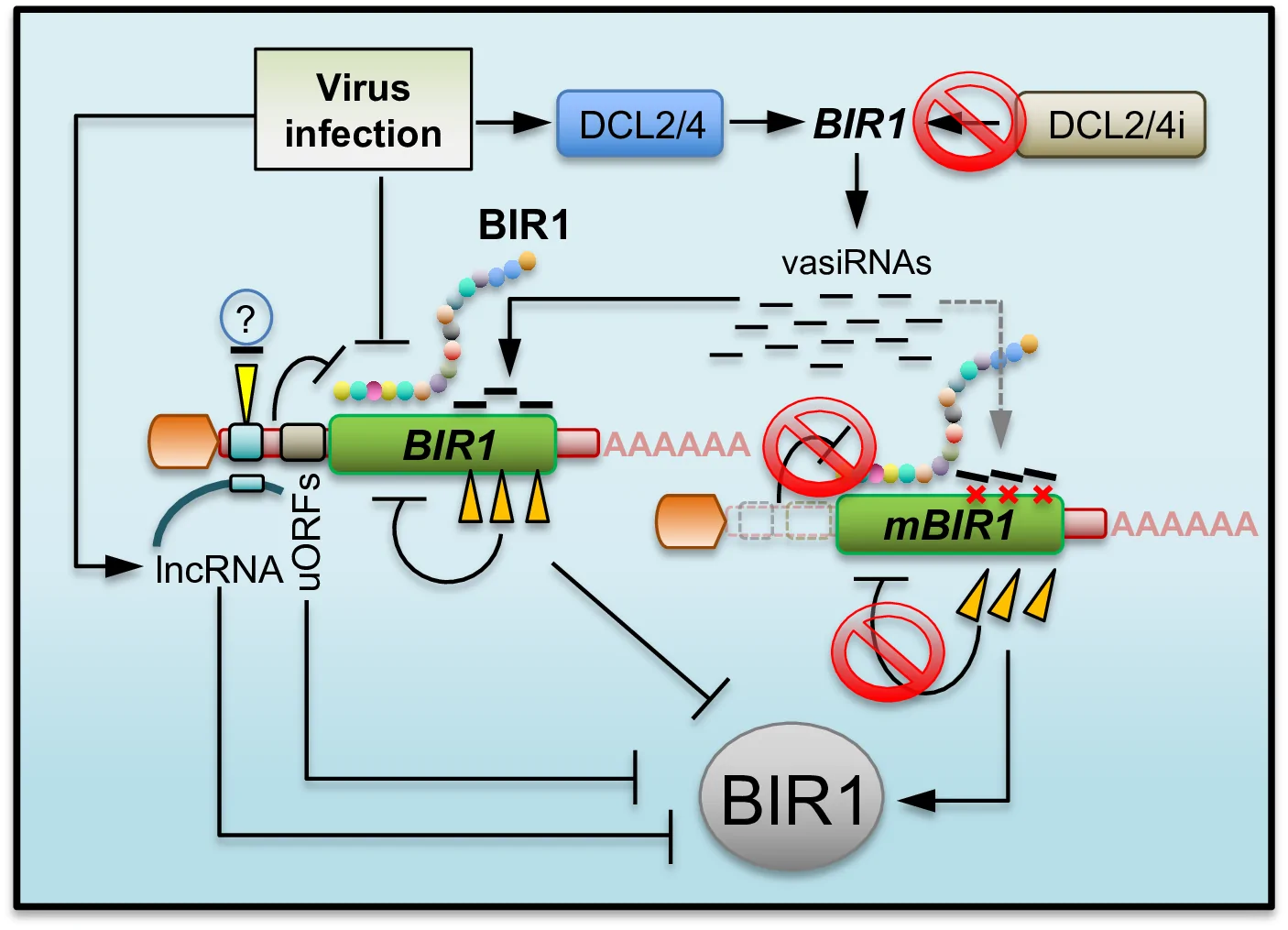
Multilayered regulation of *BIR1.* Proposed model integrating virus-associated small interfering RNA (vasiRNA)-mediated transcript cleavage with 5’-leader-dependent translational repression. Viral infection triggers the production of DCL2- and DCL4-dependent vasiRNAs that repress *BIR1* expression through site-specific cleavage of *BIR1* transcripts (orange arrows). This regulatory pathway is abolished by cleavage-site mutations in mutated *BIR1* (mBIR1) (red crosses), likely because they alter RNA structure and reduce AGO-siRNA accessibility, or in plants defective in RNA silencing (DCL2/4i). Viral infection reduces *BIR1* translation efficiency while inducing an *At4g39838*-derived long non-coding RNA (lncRNA), proposed to repress *BIR1* translation through RNA-RNA interactions with the 5’ leader region. This region also contains multiple translatable upstream open reading frames (uORFs) that likely contribute to translational regulation. Cleavage events detected within the 5’ leader (yellow arrow) may be mediated either by biologically active sRNAs or through sRNA-independent mechanisms.

In summary, our results uncover a sophisticated multilayered regulatory mechanism that integrates silencing-mediated transcript cleavage with 5’-leader-dependent translational repression to limit excess *BIR1* expression during infection (Fig 8). Tight control of *BIR1* expression is essential for immune homeostasis, and this regulatory network may therefore act independently or together with other pathways to fine-tune *BIR1* expression during transcriptional activation associated with viral or other pathogen infections. The proposed model is supported by evidence obtained from different host systems and expression platforms, but further studies will be required to determine whether all regulatory layers coordinately regulate endogenous *BIR1* during Arabidopsis infection. Whether similar mechanisms regulate other members of the *BIR* family remains to be determined.

## Materials and methods

### Plant material and virus inoculation

*Arabidopsis thaliana* and *Nicotiana benthamiana* plants were grown in controlled chambers under long-day conditions (16h day/8h night) at 22 and 24°C, respectively. Arabidopsi*s* mutants used in this study included *ago1–27, ago2–1*, *ago4–2*, *ago6–3* and *ago10–3* and the triple *dcl2–1 dcl3–1 dcl4–2* (provided James C. Carrington, The Donald Danforth Plant Center, MO, USA). T-DNA insertion Arabidopsis lines *SALK_097507C* and *SALK_093172C* were obtained from the Nottingham Arabidopsis Stock Centre (NASC). The *BIR1-mCherry* lines L6 and L9 under a DEX-inducible system were as described previously [42]. The same *BIR1-mCherry* system was used to generate *BIR1-mCherry* mutants containing nucleotide substitutions. Sequence mutations were introduced into the *BIR1* coding sequence by overlapping PCRs. These constructs were used for agroinfiltration assays and to transform Arabidopsis via floral dipping. Independent homozygous lines were selected in T3 for further experiments. Morphological phenotypes and gene expression after DEX treatments were examined in at least two independent transgenic lines. The *N. benthamiana* transgenic RNAi line NbDCL2/4i contains a chimeric hairpin construct targeting simultaneously *DCL2* and *DCL4* genes as described [75] (provided by Kriton Kalantidis, Institute of Molecular Biology and Biotechnology, Crete, Greece). The *At4g39838* genomic region containing the predicted lncRNA sequence, including the putative ath-miR5658 precursor, was amplified from Arabidopsis genomic DNA and cloned downstream of the CaMV 35S promoter in a binary pCAMBIA2300 expression vector. Homozygous T3 lines carrying single-locus insertions were identified by segregation analysis. CRISPR-Cas9-mediated mutagenesis was performed as previously described [76] to target the overlapping *At4g39838*/*At4g39840* region. Two single-guide RNAs (sgRNAs) (5’-GGAACTTGGGGGTTTTATGG-3’ and 5’- gtgAACGATAAGAGAGAAGA -3’) flanking the predicted lncRNA-binding site, including the putative miR5658 precursor region, were designed and assembled into a Cas9 expression vector carrying the FastRed selection marker using Golden Gate and Gateway cloning strategies. Arabidopsis plants were transformed by floral dipping, and transgenic lines were screened by PCR and Sanger sequencing. The selected homozygous T3 line carried a 698-bp deletion spanning the candidate region. Primers are listed in S3 Table.

The Arabidopsis *BIR1* genomic sequence (*At5g48380*) annotated in TAIR11 was PCR-amplified and cloned into the pGWB14 binary vector or the modified pYL156 vector, both carrying a C-terminal 3xHA tag. The 5’-leader sequence of *BIR1* was inserted upstream of the green fluorescence protein (GFP) coding region into the pGWB405 vector. *N. benthamiana* plants were agro-inoculated with a TRV infectious clone expressing green fluorescent protein (GFP) or *BIR1* derivatives under the replicase promoter of the pea early browning virus as described [77]. Arabidopsis plants were mechanically inoculated with TRV derivatives using sap from upper, non-inoculated leaves of *N. benthamiana* plants. Sap was prepared by grinding infected tissue in 0.5M phosphate buffer (pH 7.0) at a 1:1 (W/V) ratio and applied onto the leaf surface using carborundum as an abrasive. Relative TRV levels in *N. benthamiana* plants were measured on upper non-inoculated leaves 5 days post-inoculation (dpi) using RT-qPCR.

### Transient expression in *N. benthamiana*

Transient expression was conducted in *N. benthamiana* plants grown under long day conditions (16h day/8h night at 25°C). Agroinfiltration of *N. benthamiana* leaves was performed as described previously [78]. In co-infiltration assays, cultures containing the viral constructs were infiltrated at OD_600_ of 0.2, cultures harboring lncRNA or miRNA precursor constructs at OD_600_ of 0.4 and cultures carrying *BIR1* derivatives were infiltrated at OD_600_ of 0.4. The final concentration of bacteria was normalized within each experiment by varying the concentration of cells containing empty vector.

### RNA analysis and quantitative RT-PCR assay

Total RNA was extracted using TRIzol reagent (Invitrogen). Denaturing gel blot hybridization of normalized total RNA was performed as previously described [79]. Radiolabeled DNA probes were synthesized by random priming with [α-³²P]-dCTP. One-step qRT-PCR was conducted using Brilliant III Ultra-Fast SYBR Green QRT-PCR Master Mix (Agilent Technologies) on a Rotor-Gene 6000 or Rotor-Gene Q real-time PCR system (Corbett/QIAGEN), following [80]. Relative gene expression was calculated using the ΔΔCt method with Rotor-Gene software. Expression was normalized to *CBP20* (*At5g44200*), which showed stable expression in both mock- and virus-infected samples. Primers used are listed in S3 Table.

### Protein analysis

Leaf tissue was ground in liquid nitrogen and homogenized in extraction buffer (65 mM Tris-HCl, pH 8.0; 3% SDS; 1% β-mercaptoethanol; 10% glycerol). Samples were mixed with Laemmli buffer, heated at 95 °C for 5 min, and separated on 10% SDS-polyacrylamide gel electrophoresis (PAGE) gels. Proteins were transferred to P0.45 Polyvinylidene Fluoride (PVDF) blotting membranes (Amersham) and detected using Horseradish Peroxidase (HRP)-conjugated secondary antibodies and chemiluminescent substrate (LiteAblot Plus).

### Small RNA sequencing, construction of degradome libraries and 5’ RACE

Young rosette leaves from virus-infected and corresponding mock-inoculated plants (10–12 per sample) were pooled at 8 dpi (TRV) or 14 dpi (Turnip mosaic virus, TuMV) for degradome or sRNA sequencing. Infection was confirmed in each plant, and mock-inoculated controls were verified to be virus-free. Tissue was flash-frozen in liquid nitrogen and stored at –80 °C until use. Total RNA was extracted using TRIzol reagent (Invitrogen) or the RNeasy Plant Mini Kit (QIAGEN), and RNA quality was assessed with an Agilent 2100 Bioanalyzer. sRNA libraries were prepared and sequenced as previously described [42].

Arabidopsis degradome analysis was performed using two complementary high-throughput methods. First, differentially processed transcripts were identified via T7 RNA polymerase-mediated amplification of 5’-cleaved RNAs and hybridization to ATH1 microarrays (~24,000 transcript variants from ~22,500 genes), following the protocol by [47]. Three independent biological replicates and their respective negative controls (cDNA with RNA adapter present but not ligated) were hybridized independently. Genes with ≥2-fold enrichment and FDR ≤ 0.05 were considered significantly overrepresented. In parallel, degradome libraries were prepared from mock- and virus-infected plants for high-throughput sequencing using the PARE method, as described (Guzmán-Benito et al., 2019). Internal cleavage sites on predicted degradation targets were validated by 5’-RACE [81].

### Polysome RNA extraction

Extraction of polysome RNA was performed as described with slight modifications [82]. Briefly, individual 3-week-old mock-treated and TRV-infected Arabidopsis rosettes were flash-frozen and ground to a fine powder in liquid nitrogen. Each rosette was resuspended in 4 mL of Polysome Extraction Buffer (200 mM Tris-HCl [pH 9], 200 mM KCl, 200 mM Sucrose, 25 mM EGTA [pH 8], 35 mM MgCl2, 1.25% detergent mix [20% (w/v or v/v) of each of these four detergents in water: Brij-35, Triton X-100, Igepal CA 630 and Tween 20], 5 mM DTT, 100 µg/ml cycloheximide, and 50 µg/ml chloramphenicol). Extracts were filtered through three layers of Miracloth and centrifuged at 7,500 × g for 10 min. The supernatants were loaded onto 450 µL of a Sucrose Cushion (1,75 M sucrose, 400 mM Tris-HCl [pH 9], 200 mM KCl, 5 mM EGTA, 25 mM MgCl_2_, 5 mM DTT, 50 µg/ml cycloheximide, and 50 µg/ml chloramphenicol) and centrifuged at 40,000 rpm in a Hitachi P40ST rotor for 3.5 h, at 4 °C. The supernatants were discarded, and the pellets were briefly washed with ice-cold distilled water and resuspended in 300 µL of Polysome Resuspension Buffer (200 mM Tris-HCl [pH 9], 200 mM KCl, 25 mM EGTA [pH 8], 35 mM MgCl_2_, 5 mM DTT, 100 µg/ml cycloheximide, and 50 µg/ml chloramphenicol). RNA was extracted following the SDS/acid phenol method [83], followed by LiCl precipitation.

### RT-qPCR analysis for the calculation of the translation efficiency of *BIR1*

*BIR1* expression was measured in total and polysomal RNA in mock-treated and TRV-infected 3-week-old Arabidopsis plants. Total RNA and polysomal RNA were DNAse-treated (TURBO DNase Ambion) and reverse-transcribed (iScript Bio-Rad) following manufacturers’ recommendations. cDNA was used as a template for real-time qPCR using SSoFast Eva Green Supermix (Bio-Rad). *CBP20* was used as the reference gene. Primers used are listed in S3 Table.

### Analyses of upstream Open Reading Frames (uORF)

Ribo-seq and RNA-seq datasets from Col-0 plants under control and ETI-induced conditions were obtained from Zhou *et al*. (2023) and analyzed following the procedures described therein. Reads were aligned to *BIR1*, and normalized read counts were quantified for the main open reading frame (mORF) and annotated upstream open reading frames (uORFs). Ratios of read counts were calculated between experimental conditions and between the mORF and uORFs.

### Data access

Arabidopsis genome annotations (TAIR11) were obtained from TAIR (www.arabidopsis.org). Degradome data from naïve Col-0 inflorescences (GSM280226) were previously published [84]. Data from this study are available in the NCBI Gene Expression Omnibus (GEO) under the following accession numbers: GSE106322 (Microarray hybridization of degradome tags), GSM3019138–40 (Deep sequencing of degradome tags), GSM2808011–12, GSM3019141–42 (Deep sequencing of sRNAs).

## Supporting information

S1 Fig Mutations at predicted cleavage sites within the *BIR1* coding sequence enhance transcript accumulation. (A) RT-qPCR of TRV RNA 1 in *Nicotiana benthamiana* upper leaves at 5 days post-infiltration (dpi), systemically infected with TRV derivatives expressing GFP, 5’-leader-containing *BIR1* variants (WT, mA-1, mA-2, mA-3, mBCD) or *BIR1* variants lacking the 5’-leader (Δ5’ UTR) (WT, mBC, mBCD). (B) RT-qPCR analysis of *BIR1-mCherry* transcripts in DEX-inducible transgenic Arabidopsis lines expressing wild-type (WT: L6, L9) or mutated (mBCD: L3, L7) BIR1 after 6 and 24 h of DEX treatment (30 µM). All RT-qPCR data were normalized to *CBP20*. Data represent mean ± SD (n = 3) and were analyzed by one-way ANOVA followed by Tukey’s multiple-comparison test.
(TIF)

S2 Fig Characterization of regulatory elements within the 5’-leader of *BIR1.* (A) Schematic of the *At4g39838* locus and T-DNA insertion sites (SALK_097507C, SALK_093172C) shown as dotted triangles; transcription start sites as black arrows. (B) RT-PCR of lncRNA transcripts derived from the *At4g39838* locus in WT and T-DNA lines, with (+) or without (–) reverse transcriptase. β-Tubulin was used as a control. (C) Predicted ath-miR5658 interaction sites within the TCA-rich 5’ untranslated leader region of *BIR1*. G-U base pairs (black dots) and mismatches (red dots) are indicated. The major cleavage site A at position 156 (from degradome sequencing) is marked with a thick arrow. (D) Predicted secondary structure of the pre-miR5658 RNA. The mature ath-miR5658 sequence is highlighted within the stem-loop. (E) RNA blot of small RNAs (sRNAs) from leaves infiltrated with 35S-driven pre-miR5658 constructs at 2 days post-infiltration (dpi). Blots were probed with A (ath-miR5658-complementary) and B/C (outside the pre-miR5658 region) as shown. Empty vector (EV) was used as control. A 21-nt size marker is indicated. (F) Predicted interaction between a BIR1-targeting sRNA and the TCA-rich 5’ leader. (G) Arabidopsis loci matching the candidate BIR1-interacting sRNA and virus-associated siRNA (vasiRNA) production in mock- and virus-infected plants (tobacco rattle virus (TRV), left; turnip mosaic virus (TuMV), right).
(TIF)

S3 Fig Identification of *BIR1*-interacting virus-associated small RNAs (sRNAs) in Arabidopsis. (A) Distribution of sRNAs derived from *At4g39838* in mock- and turnip mosaic virus (TuMV)- or tobacco rattle virus (TRV)-infected Arabidopsis plants. The shaded region marks the natural antisense overlap between *At4g39838* and *At4g39840*. (B) Distribution of sRNAs derived from *At1g42990* in mock- and TuMV- or TRV-infected plants. The shaded region represents the *At1g42990* coding sequence. Sense (black dots) and antisense (red dots) sRNAs are plotted as positive and negative values, respectively. N indicates the total number of filtered sRNA reads.
(TIF)

S1 Table Potential RNA silencing target genes detected in mock-inoculated and TRV-infected inflorescences. (XLSX)

S2 Table Relative expression levels of RNA silencing-related genes in TRV-infected leaves (8 days post-inoculation, dpi), inflorescences (12 dpi) and roots (12 dpi) relative to the same tissues from mock-treated Arabidopsis. (XLSX)

S3 Table List of primers. (XLSX)
